# Unified frame of reference improves inter-subject variability of seismocardiograms

**DOI:** 10.1186/s12938-015-0013-9

**Published:** 2015-02-26

**Authors:** Mikko Paukkunen, Petteri Parkkila, Raimo Kettunen, Raimo Sepponen

**Affiliations:** Department of Electrical Engineering and Automation, Aalto University, P.O. BOX: FI-13340, 00076 Helsinki, Finland; School of Medicine, University of Eastern Finland, Kuopio, Finland; Health Factory, Aalto University, P.O. BOX: FI-13340, 00076 Helsinki, Finland

**Keywords:** Seismocardiography, Ballistocardiography, Aaccelerometer, Three-dimensional, Inter-subject variability, Frame of reference

## Abstract

**Background:**

Seismocardiography is the noninvasive measurement of cardiac vibrations transmitted to the chest wall by the heart during its movement. While most applications for seismocardiography are based on unidirectional acceleration measurement, several studies have highlighted the importance of three-dimensional measurements in cardiac vibration studies. One of the main challenges in using three-dimensional measurements in seismocardiography is the significant inter-subject variability of waveforms. This study investigates the feasibility of using a unified frame of reference to improve the inter-subject variability of seismocardiographic waveforms.

**Methods:**

Three-dimensional seismocardiography signals were acquired from ten healthy subjects to test the feasibility of the present method for improving inter-subject variability of three-dimensional seismocardiograms. The first frame of reference candidate was the orientation of the line connecting the points representing mitral valve closure and aortic valve opening in seismocardiograms. The second candidate was the orientation of the line connecting the two most distant points in the three dimensional seismocardiogram. The unification of the frame of reference was performed by rotating each subject’s three-dimensional seismocardiograms so that the lines connecting the desired features were parallel between subjects.

**Results:**

The morphology of the three-dimensional seismocardiograms varied strongly from subject to subject. Fixing the frame of reference to the line connecting the MC and AO peaks enhanced the correlation between the subjects in the y axis from 0.42 ± 0.30 to 0.83 ± 0.14. The mean correlation calculated from all axes increased from 0.56 ± 0.26 to 0.71 ± 0.24 using the line connecting the mitral valve closure and aortic valve opening as the frame of reference. When the line connecting the two most distant points was used as a frame of reference, the correlation improved to 0.60 ± 0.22.

**Conclusions:**

The results indicate that using a unified frame of reference is a promising method for improving the inter-subject variability of three-dimensional seismocardiograms. Also, it is observed that three-dimensional seismocardiograms seem to have latent inter-subject similarities, which are feasible to be revealed. Because the projections of the cardiac vibrations on the measurement axes differ significantly, it seems obligatory to use three-dimensional measurements when seismocardiogram analysis is based on waveform morphology.

## Background

Seismocardiography (SCG) is the noninvasive measurement of cardiac vibrations transmitted to the chest wall by the heart during its movement [1]. The emergence of SCG measurement can be traced back to 19th century when Gordon reported observing a heartbeat while standing on a scale [2]. Gordon’s observation led off the development of several measurement methods for cardiac induced vibrations such as ballistocardiography (BCG). BCG is a method in which the reaction forces acting on the whole human body are measured. Thus, SCG can be viewed as a derivative of BCG. The emergence of SCG as a separate measurement technique dates back to 1960s when the pioneering works of Baevsky and Bozhenko [3,4] were published. The advances in electronic accelerometer technology have resulted in multiple applications for SCG, such as ischemic heart disease detection, gating of cardiac imaging and therapy, as well as smartphone-based heart activity monitoring [5-9].

Most applications for SCG are based on unidirectional acceleration measurement, usually done in the back-to-front direction. However, several studies have highlighted the importance of three-dimensional (3-D) measurements in cardiac vibration studies [10-13]. For example, Migeotte et al. concluded that the vibration waveforms observed on the foot-to-head axis are poorly correlated to the magnitude of the maximum systolic force vector computed using all three axes of acceleration [10]. This finding indicates that the vibrations of the heart might be projected disproportionately between the three orthogonal axes of measurement and may thus lead to misinterpretation of the waveforms and the underlying physiological phenomena. Another fairly recent paper reported a method that was based on 3-D SCG vector trajectories which were used for computing the IJ-waveform amplitudes [14]. The IJ-waveforms were proposed to be related to cardiac output. The report found that that since the 3-D approach takes into account the uneven distribution of vibrations between the measured axes, the achieved results might be independent from slight misalignments of the SCG sensor. This indicates that if slight misorientations of the SCG sensor are accountable, the compensation of inter-subject variation due to physiological differences should also be feasible.

Inter-subject variability of seismocardiograms, which hinders the deployment of SCG in to clinical practice, is a widely observed phenomenon in SCG studies [15,16]. We hypothesize that a unified frame of reference would facilitate improvement of inter-subject variability of seismocardiograms. The hypothesis relies on two assumptions. The first assumption is that asymptomatic hearts function similarly in general. Thus, only modest inter-subject variability should be seen when measuring SCG from healthy subjects. However, significant inter-subject differences in seismocardiograms are continuously reported even in healthy subjects. This indicates that SCG measurements are prone to variability due to other factors than differences in cardiac function. These other factors could be anatomical differences such as the orientation of the heart and the aortic arc or differences in the mechanical coupling of the myocardial vibrations to the sternum. The second assumption is that 3-D acceleration measurement captures the vibrations of the chest sternum in every direction. Thus, even if the vibrations of the heart are projected differently between subjects, the use of a unified frame of reference might straighten the vector trajectories and reveal latent similarity in the seismocardiograms.

This study investigates the feasibility of using a unified frame of reference to improve the inter-subject variability of SCG waveforms. To test the feasibility of the proposed method, a data set from ten subjects is acquired and pre-processed.

## Methods

### Equipment

The 3-D SCG signals were measured with three orthogonally mounted accelerometers (SCA610-C21H1A, Murata Electronics, Finland). The accelerometers and associated electronics are both described in detail in the authors’ previous work [17]. For this study, we mounted the individual accelerometers to a smaller package than before using eyesight to orient the sensors. Due to the mounting process, the orthogonality of the acceleration sensors could not be guaranteed. The largest orientation error between the accelerometers was measured between the xy plane and the z axis (5 degrees). Thus, in the worst case, any single axis of acceleration measurement will include an error of 9% compared to ideally orthogonal accelerometers. The acceleration sensors had a sensitivity of 2 V/g, true DC response and rated output noise of approximately 60 μg_rms_ in the frequency band of 1 to 50 Hz. Prior to use, the accelerometers were tested at VTI’s (now Murata Electronics) laboratories where they were shown to have their -3 dB points at 47 Hz. The acceleration signals were anti-alias filtered with 8th-order Bessel lowpass filters with a rated cut-off frequency of 100 Hz and attenuation of at least 96 dB at 800 Hz. The filters are coupled in the Sallen-Key topology using quad operational amplifiers (AD8630, Analog Devices, USA). For this particular study, the gain stage of the acceleration measurement in [17] is bypassed, resulting in a DC response and a gain of 0 dB for the acceleration measurement. Three individual accelerometers were used because, to the best of the authors’ knowledge, no integrated options with similar characteristics (high sensitivity, low noise, DC response) were available during the time of implementation. In terms of accuracy, there is no inherent benefit in using individual accelerometers compared to integrated 3-D accelerometers. In particular, recent research has applied integrated 3-D accelerometers with similar noise and sensitivity performance to SCG but with no DC response [18]. A DC response would be important in other SCG applications where the instantaneous inclination of the sternum was of interest.

The electrocardiography (ECG) lead II was measured using a commercial wireless dual-lead ECG system (BC-ECG2, BIOPAC Systems Inc, US). Respiratory efforts were detect using a commercial wireless respiratory effort detection system (BN-RESP-XDCR, BIOPAC Systems Inc, US). All data were captured using the MP150 Data Acquisition System (BIOPAC Systems Inc, US). The acceleration signals were coupled to the MP150 through a Universal Interface Module (UIM100C, BIOPAC Systems Inc, US). Preprocessing of the signals was done in the Acknowledgment environment (BIOPAC Systems Inc, US). All post-processing of the data was done in the Matlab environment (MATLAB R2013b, Mathworks, US).

### In vivo measurements

#### Human subject protocol

The present study was completely conducted in the premises of Aalto University, Espoo, Finland. Ten male volunteers with an average age of 33 years (standard deviation (SD) 8.5 years), an average weight of 79.5 kg (SD 14.9 kg) and an average height of 175.2 cm (SD 7.1 cm) were measured as test subjects. During the measurements, the subjects were at resting supine position. To minimize breathing-induced variability, the subjects were instructed to hold their breath as long as comfortable.

**Figure 1:**
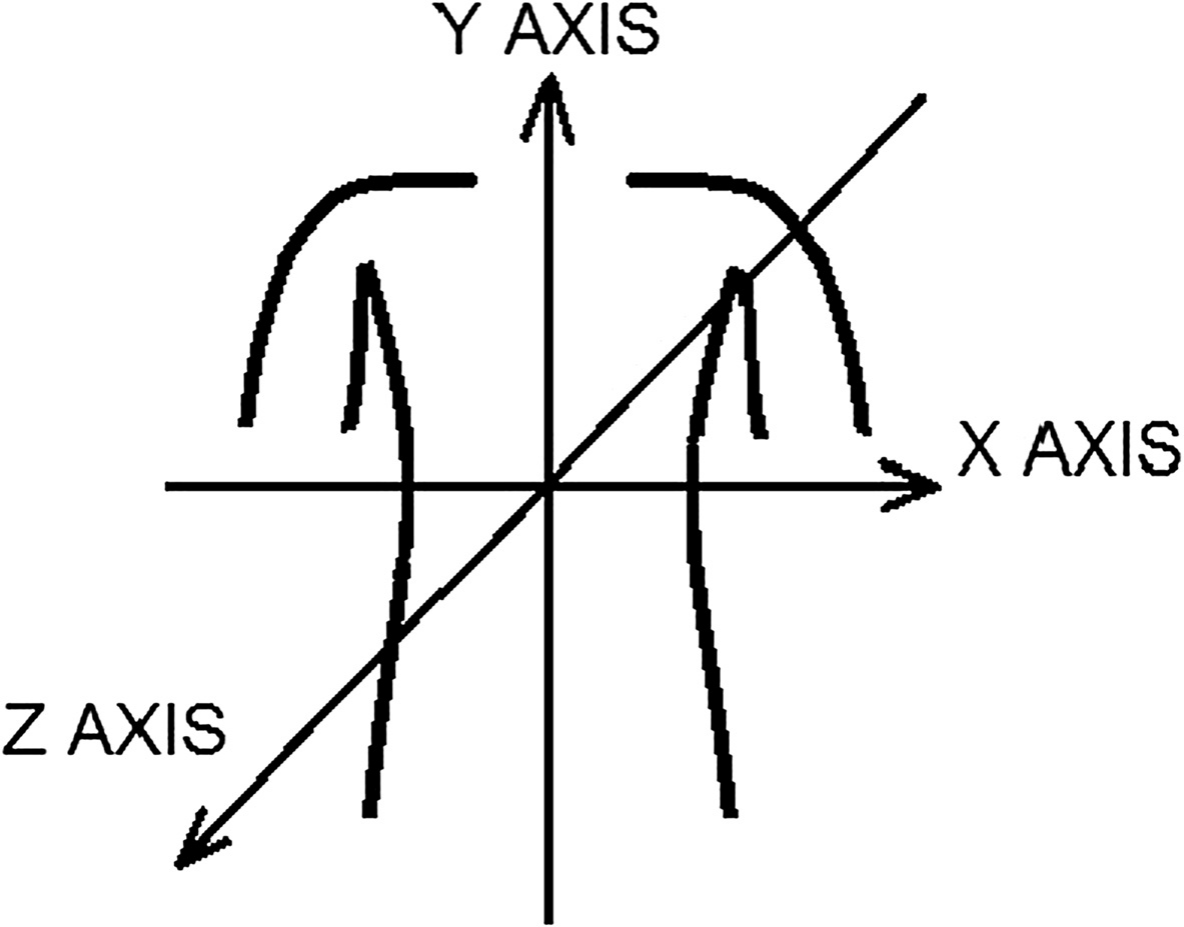
**Measurement axes. **The x axis is the left-to-right direction, the y axis the foot-to-head direction and the z axis the back-to-front direction.

The accelerometer package was positioned on the lower part of the sternum about one centimeter above the xiphoid process using double-sided adhesive tape. The accelerometer package was mounted so that one accelerometer measured the acceleration in the back-to-front (z axis), one in the right-to-left (x axis), and one in the foot-to-head direction (y axis) (see Figure 1).

The contents of the test and the course of its events were explained individually to the subjects before the study. In addition, a written consent was received. The study did not contain any such intervention in the physical integrity of the test subjects, or any other features that would require an ethical review as considered by the National Advisory Board on Research Ethics in Finland.

### Selecting the frame of reference

The rationale behind using a unified frame of reference lies in the assumption that most hearts function similarly in general. Thus, the inter-subject differences might be mainly due to anatomical differences and differences in the mechanical coupling of the heart’s vibration to the sternum. Some methods that might be useful in managing the variability of seismocardiograms have been presented in the literature. The approaches include the division of heartbeats in to inspiration and expiration heartbeats [19] and normalization by resampling each heartbeat to be of same length [13], for example. Castiglioni et al., for example, proposed that the magnitude of the IJ-waveform computed from 3-D seismocardiograms is independent of slight orientation differences of the SCG sensor [14]. However, the use of orientation information, which is readily obtainable from 3-D SCG signal, is rarely reported.

**Figure 2:**
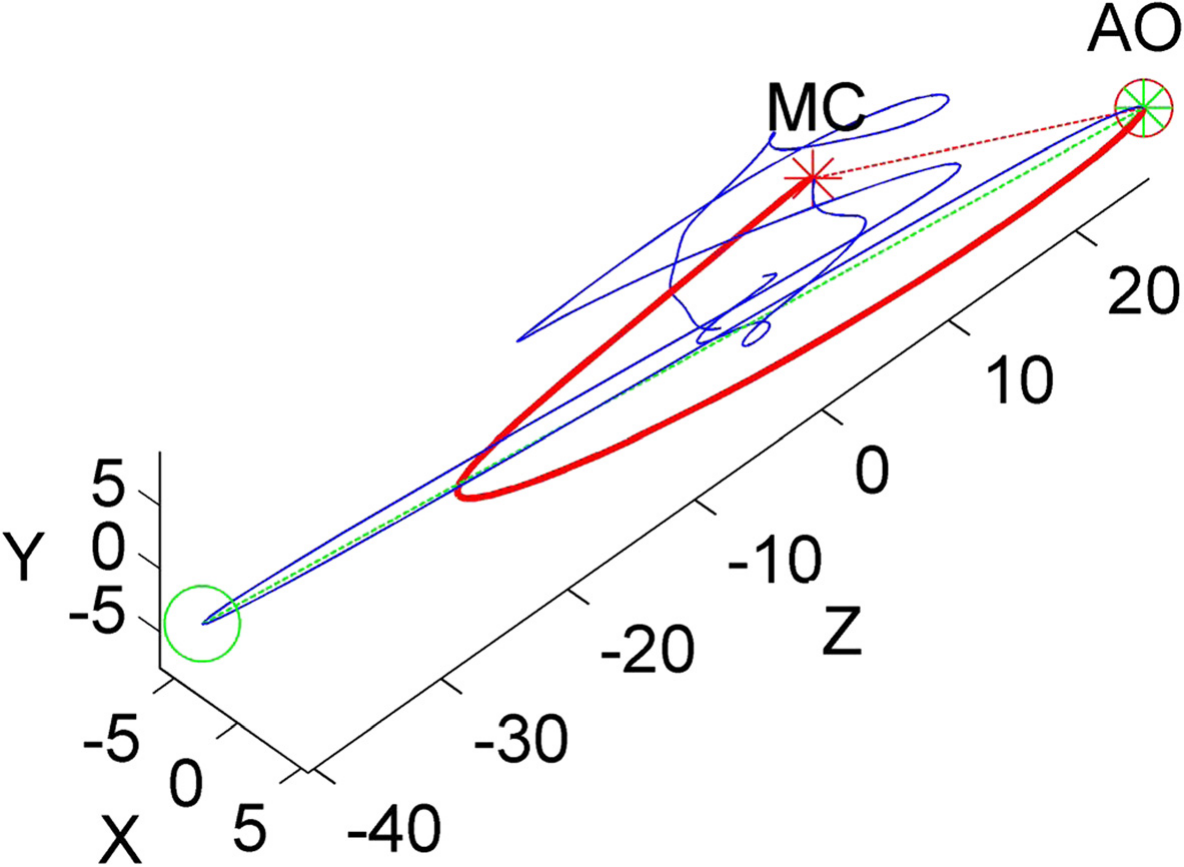
**Frames of reference. **The red asterisk marks the MC point and the green asterisk the AO point. The green and red circles are the two most distant points in the seismocardiogram. The red dashed line connects the MC and AO points, and the green dashed line connects the two most distant points in the seismocardiogram. The units are in milli-gs (1 mg = 9,81 mm/s^2^).

The authors hypothesize that if some orientation-related representative feature could be extracted from every seismocardiogram, this feature could be used to normalize the orientation of seismocardiograms so that each seismocardiogram could be interpreted in the same frame of reference. Thus, this approach would facilitate reducing the effect of mechanical coupling and anatomic differences in the seismocardiograms. Two candidates were selected to serve as frames of reference. The first candidate was the orientation of the line connecting the points that represent mitral valve closure (MC) and aortic valve opening (AO) (see Figure 2). During this time interval, the ventricles contract with no change in volume (i.e. isovolumic contraction) and no blood flows to the aorta. Thus, only respiration efforts and the movement of the heart affect the SCG waveforms. As the baseline shifts in SCG signal due to respiratory efforts (i.e. the inclination of the sternum changing) can be effectively eliminated with high-pass filtering, the orientation of the filtered SCG signal during isovolumic contraction can be assumed to depict the orientation of the heart’s mechanical axis. Recent research has suggested that breathing manifests also in SCG morphology [20]. In this study, we attempt to minimize these changes by restricting the analysis to epochs of breath holding. The second candidate was the orientation of the line connecting the two most distant points in the 3-D seismocardiogram (see Figure 2). This line was assumed to represent the direction in which the heart could produce maximal systolic force.

### Signal processing

#### Pre-processing

All SCG and ECG signals were digitally filtered in BIOPAC with a band-pass filter using a 0.5 Hz lower and a 40 Hz upper cut-off frequency. Based on the respiratory signal, the data where the breath hold takes place was manually selected for each subject. R peaks were detected using a simplified Pan-Tompkins algorithm [21] by first finding all cardiac cycles with an arbitrary length and then seeking the maximum values inside each cycle. Extraction of SCG cycles was then performed by segmenting the signals to 700-ms long windows starting 100 ms before and ending 600 ms after each ECG R peak. We found that the timings of the relevant SCG features in relation to the R peaks were not associated with variable heart rate, and thus normalization would have drastically affected the averaging process. Therefore, in this study, no heart rate normalization was performed. After segmentation, ensemble averaging was done by using the largest number of heartbeats that was available from all subjects (11 beats). These heartbeats were selected by minimizing the mean standard deviation of the resulting ensemble averages. MC and AO peaks were manually detected from averaged z axis SCG signals using the annotation scheme proposed by Crow et al. as a guideline [22]. The MC point was identified as the positive peak on the z-axis seismocardiogram following the onset of the QRS complex, whereas the AO point is the positive peak after the MC point.

### Computing the frame of reference

**Figure 3:**
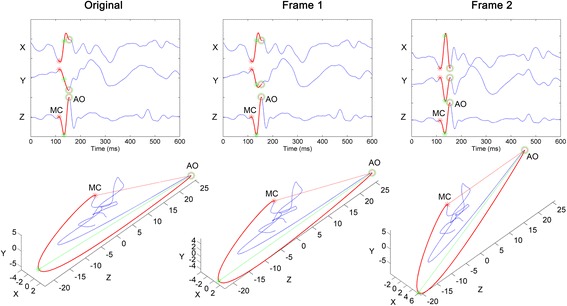
**Rotation of seismocardiograms.** The leftmost column shows the original unrotated signal of a subject, the plots in the middle show the original signal rotated so that the line connecting the two most distant points is parallel to the z axis (frame 1), and the rightmost column shows the original signal rotated so that the line connecting the MC and AO points is parallel to the z axis (frame 2). The units are in mg.

A 3-D seismocardiogram is defined as a vector function of time with magnitude of $\sqrt{X^2+{Y}^2+{Z}^2}$ where each of the SCG axes represents a Cartesian coordinate in 3-D space. Ensemble averaged 3-D seismocardiograms for each subject were rotated separately based on the two frames of reference, which were the line connecting the most distant points (Frame 1) and the line connecting the MC and AO peaks (Frame 2), as described previously. To unify the orientation of the original 3-D seismocardiograms, they were rotated so that either frame 1 or frame 2 was parallel with the z axis (Figure 3). Rotation was performed in a plane with a normal vector described by the cross product of the frame of reference and the z axis.

### Evaluation of the inter-subject variability of seismocardiograms

Squared Pearson correlation coefficients (R^2^) as well as root-mean-square error (RMSE) and normalised root-mean-square error (NRMSE) in the MC-AO interval for each SCG axis were calculated in order to quantify the inter-subject variability in the 3-D seismocardiograms both before and after the rotations. For each SCG axis, this procedure resulted in ${\displaystyle \sum_1^{10 - 1}}n=45$ different correlation coefficients, RMSE, and NRMSE values between the ten subjects.

## Results and discussion

**Figure 4:**
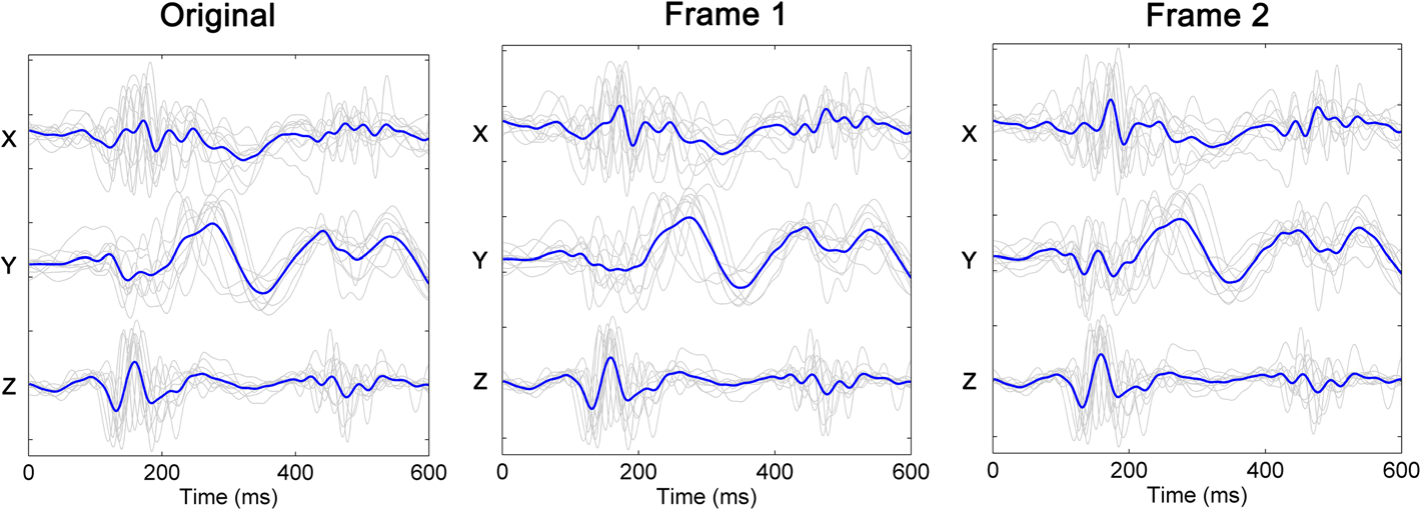
**Effect of rotation on individual seismocardiogram trajectories.** The individual seismocardiogram trajectories without rotation (left), with rotation using frame 1 (center), and with rotation using frame 2 (right) are shown. The gray plots are the mean seismocardiograms of individual subjects. The blue lines are the mean of the means of seismocardiograms of individual subjects.

The effect of rotations on the inter-subject correlation, RMSE, and NRMSE is shown in Table 1. The morphology of the 3-D SCG waveforms varied strongly from subject to subject, as evidenced by the relatively weak inter-subject correlation (mean correlation of 0.39 on the x axis, 0.42 on the y axis, and 0.86 on the z axis) of the unprocessed 3-D seismocardiograms. Fixing the frame of reference to the line connecting the MC and AO peaks (i.e., frame 2) considerably enhanced the correlation between the subjects in the y axis (mean correlation of 0.52 and 0.83 using frame 1 and frame 2, respectively). Also, the NRMSE decreased from 0.43 to 0.29 on using frame 2, suggesting the rotation process decreased the inter-subject variability. However, the impact of using a unified frame of reference was not as prominent in the other axes. The x axis correlations were only slightly improved and RMSE slightly decreased using either frame of reference. The proposed method did not significantly affect the standard SCG z-axis features. This is quantified by the high correlation between the original and rotated z axis signals (subject-wise mean of 0.99 for frame 1 and 0.95 for frame 2) as well as the consistency of the original and rotated z axis waveform RMSEs. Also, it can be visually confirmed from Figure 4 that the z-axis waveforms remain similar despite the rotation process. Using the frame 2, the mean correlation across all axes was improved from 0.56 to 0.71. Also, 44 out of 45 y axis correlation coefficients were improved using frame 2. Despite the seemingly low amount of increased coefficients in X and Z axes, well over half of the coefficients in separate axes did improve when both frames of reference were considered (31, 44 and 27 in the x, y, and z axis, respectively).

**Table 1:** **Effect of rotation**

	**RMSE [mg]**			**Normalized RMSE**			**Correlation**		
**Axis**	**x**	**y**	**z**	**x**	**y**	**z**	**x**	**y**	**z**
Unrotated	4.91±1.76	3.63±1.59	7.26±3.40	0.43±0.12	0.43±0.17	0.13±0.05	0.39±0.30	0.42±0.31	0.86±0.13
Frame 1	3.90±1.56	3.62±1.81	7.47±3.51	0.55±0.19	0.45±0.16	0.13±0.05	0.44±0.33	0.52±0.31	0.85±0.13
Frame 2	4.40±1.68	4.60±2.49	7.27±3.63	0.47±0.21	0.29±0.13	0.14±0.05	0.43±0.31	0.83±0.14	0.85±0.14

Although the y axis correlations were improved remarkably when a unified frame of reference was used, the x axis correlations remained weak. This suggests that the x axis does not contain significant physiological information. As the accelerometers used in this study were very sensitive (2 V/g), it seems that insufficient coupling of cardiac and blood flow-induced vibrations to the sternum might partly cause the weak inter-subject correlation. Migeotte et al. also observed that most of the time the cardiac function is projected almost entirely on the yz-plane [13]. To enhance 3-D SCG, the characteristics of the x axis should be further studied.

While the present method was feasible to remarkably improve the y axis correlations, the z axis correlations were virtually unaffected. The possibility of masking or modification of individual biological issues cannot, however, be excluded and it is yet unclear whether these biological issues are important. The proposed method does not wipe out information but only displays it differently. The mounting of the accelerometers might have affected the results. As the orientation error found in the accelerometers was relatively small, the effect of this error might be negligible. However, the degree of the effect must be quantified in future research. Also, we suggest that in future work the accelerometers should be mounted using a repeatable process that will guarantee the orthogonality of the accelerometers.

The z axis SCG has been proposed as feasible to be used in cardiac time interval measurements [23]. Given the high z axis correlations, it seems that the capability of the z axis to be used in measuring cardiac time intervals was not affected when a unified frame of reference was used. This observation further asserts the feasibility of using a unified frame of reference to improve 3-D SCG measurements.

## Conclusions

The results achieved in this study indicate that the use of a unified frame of reference is a promising method for improving the inter-subject variability of 3-D seismocardiograms. A larger sample size is needed to reassert the validity of this method. More importantly, these results show that 3-D seismocardiograms have latent inter-subject similarities, which are feasible to be revealed. Because the projections of the cardiac vibrations on the measurement axes differ greatly, it seems obligatory to use 3-D SCG measurements when analysis is based on SCG waveform morphology rather than time intervals.
